# Age-dependent changes in the expression and localization of LYZL4, LYZL6 and PCNA during testicular development in the Ashidan yak

**DOI:** 10.1080/10495398.2024.2344213

**Published:** 2024-04-26

**Authors:** Yongfu La, Zhongbang Li, Xiaoming Ma, Pengjia Bao, Min Chu, Xian Guo, Chunnian Liang, Ping Yan

**Affiliations:** a Animal Science Department, Lanzhou Institute of Husbandry and Pharmaceutical Sciences, Chinese Academy of Agricultural Sciences, Lanzhou, PR China; b Key Laboratory of Animal Genetics and Breeding on Tibetan Plateau, Ministry of Agriculture and Rural Affairs, Chinese Academy of Agricultural Sciences, Lanzhou, PR China; c Key Laboratory for Yak Genetics, Breeding, and Reproduction Engineering of Gansu Province, Chinese Academy of Agricultural Sciences, Lanzhou, PR China

**Keywords:** Yak, testis, lysozyme-like (LYZL) proteins, proliferating cell nuclear antigen

## Abstract

Lysozyme like 4 (LYZL4), lysozyme like 6 (LYZL6) and proliferating cell nuclear antigen (PCNA) are implicated in the regulation of testicular function, but there was no research reported available on the expression patterns of *LYZL4*, *LYZL6* and *PCNA* genes at different developmental stages of yak testes. In this study, we used the qRT-PCR, western blotting and immunohistochemistry estimated the *LYZL4*, *LYZL6* and *PCNA* gene expression and protein lo-calization at different developmental stages of yak testes. The qPCR results showed that the mRNA expression of *LYZL4*, *LYZL6* and *PCNA* genes significantly increased with age in the testes of yaks. Western blot results showed that the protein abundance of LYZL4, LYZL6 and PCNA in yak testes was significantly higher after puberty than before puberty. Furthermore, the results of immunohistochemistry indicated that LYZL4, LYZL6 and PCNA may be involved in the regulation of spermatogonia proliferation and Leydig cell function in immature testis. In adult yak testes, LYZL4, LYZL6 and PCNA may involve in the development of round spermatids and primary spermatocytes during testicular development. Our results indicated that LYZL4, LYZL6 and PCNA may be involved in the development of Sertoli cells, Leydig cells and gonocytes in yak testes.

## Introduction

The yak is an iconic symbol of Tibet and is found at altitudes. More than 14 million yaks provide the basic resources including meat, manure for fuel, milk, transportation and hides for tents that are necessary for nomadic pastoralists in high altitude environments.^1–3^ In the modern yak breeding and production industry, artificial insemination (AI) technology is widely used to improve the overall performance of the yak breeds.^4^ However, the male yaks mature sexually later and their testicles grow relatively slowly until approximately 30 months of age reached the sexual maturity. Therefore, the fertility of the male yak is one of the first limiting factors to achieving the highest reproduction possible. However, the testis is one of the most important reproductive organs in males, and its growth and development are regulated by the hypo-thalamic-pituitary-gonadal axis.^5^^,^^6^ The testes mainly have endocrine and exocrine functions. The exocrine function involves the production of highly differentiated gametes, the spermatozoa, released into the lumen of the seminiferous tubules and then stored in the epididymis. The endocrine function is performed by the Leydig cells, located in the interstitial compartment, outside the tubules, the main role of which is the production of steroids. Therefore, an in-depth understanding of the molecular mechanisms of testis development and testicular steroidogenesis is important for the study of reproduction in yak.

Lysozyme like 4 (LYZL4) and lysozyme like 6 (LYZL6, also known as LYC1) are members of the lysozyme like (LyzL) family and have been used to serve as biomarkers for male fertility.^7–9^ LYZL4 is a sperm bound protein located in the intra-acrosomal region of spermatozoa and the principal piece of the sperm tail.^10^ It plays an important role in the process of fertilization and is expressed in the epididymis and testis.^11^ Lyzl6 is a new c-type lysozyme-like gene, and its pattern of expression is testis/epididymis specific.^8^ It is reported that LYZL6 was present in testis, epididymis and spermatozoa of mice.^12^ In humans, *LYZL4* and *LYZL6* genes have been successfully cloned from the male reproductive system, and *LYZL4* and *LYZL6* genes were found to be expressed in the male reproductive tract.^13^^,^^14^ Proliferating cell nuclear antigen (PCNA) is a central cell protein located at the core of a complex protein network mediating DNA repair and replication, chromatin remodeling and epigenetics through interactions promoted by short linear motifs.^15–17^ PCNA is a cell cycle regulatory protein marker, is closely related to DNA synthesis and involved in the proliferation and differentiation of spermatogonia in the testis.^18^ Studies have shown that the protein expression level of PCNA in the testis is a standard marker for the proliferation of spermatogenic cells, and can be also used to assess the state of spermatogenic cell proliferation.^19–21^ The results of all these previous studies indicate that *LYZL4*, *LYZL6* and *PCNA* genes have important functions in male reproduction. However, few studies have investigated the effects of *LYZL4*, *LYZL6* and *PCNA* genes on the development of yak testes.

In this study, the regulation of *LYZL4*, *LYZL6* and *PCNA* genes in the development of yak testis and spermatogenesis was focused by testicular histology and morphology, as well as mRNA expression, protein abundance and immunohistochemical analysis of three candidate genes. Therefore, the overall goals of this study were to look at the potential role of *LYZL4*, *LYZL6* and *PCNA* genes in testicular development and spermato-genesis of yak, which can provide an insight into the application of LYZL4, LYZL6 and PCNA as uncertain markers in the yak reproduction programme.

## Materials and methods

### Ethics  statement

The study is supported by the ethical guidelines of Lanzhou Institute of Husbandry and Pharmaceutical Sciences and met the requirement of the institutional animal care and use committee (Permit No. 2019-002).

### Animals

All animals used in this study were from nucleus herds of Ashidan yak in the Datong Breeding Farm of Qinghai province. The twelve selected Ashidan yak were healthy and fed in an outdoor setting under similar conditions of temperature, illumination and nutrition level. Furthermore, the animals were separated into four groups (6 months, 18 months, 30 months and 72 months). Every group contained three male yaks. The 12 male yaks were slaughtered and tissues from the left testes were collected. Each testis was separated into two parts from the centrally: one part of the sample was immediately frozen in liquid nitrogen, and the other one was fixed in 4% formalin buffer for 48 h prior to paraffin embedding.

### Gene expression of LYZL4, LYZL6 and PCNA in the testis

Total RNA from Ashidan yak testis was isolated using the TRIzol reagent (TaKaRa, Dalian, Beijing, China). The NanoDrop spectrophotometer (Thermo Scientific, Waltham, Massachusetts) was used to estimate the concentration and quality of extracted total RNA. Subsequently, the RNA samples were reverse transcribed to cDNA using the reverse cDNA transcription kit (TaKaRa, Dalian, Beijing, China).

LYZL4, LYZL6 and PCNA primers (exon-span) for qRT-PCR were designed according to NM_001077959.1, NM_001046466.1 and XM_005906528.2, using Primer-BLAST (NCBI). The PCR reaction was performed on the LightCycler 480II (Roche, Basel, Sweden) using the SYBR Green Real-time PCR Master Mix (TOYOBOCO, LTD, Osaka, Japan) with different cycling conditions as 95 °C for 10 min, followed by 45 cycles for 15 s at 95 °C, annealing for 60 s at 55–60 °C, extension for 30 s at 72 °C, final extension for 5 min at 72 °C and storage at 4 °C. GAPDH was used as an internal reference to normalize target gene expression. All experiments were performed in triplicate. The primers (Table 1) were produced by Shenggong Biotech Co., Ltd. (Shanghai, China).

**Table 1.** Details of primer sequences used for qRT-PCR.

Primer name	Primer sequence	Product size (bp)
LYZL4	F: ACCCTATGGCTGTCTATGAA R: TGGATTCAGTAAAGCAGAGC	131
LYZL6	F: GTGCCTGGCTTTCGTAGAGA R: CGTGCGACTCTGGTAATCGT	127
PCNA	F: GTGTCATTGCGACTCCG R: AACACTGCCTACAACG	121
GAPDH	F: GGGGAAAAGCGGACTTAGGA R: TGGTTCACGCCCATCACA	143

### Western blotting

The proteins were extracted from the testis following a previously established procedure.^2^ The protein was fixed in 12% Tricine SDS-PAGE for gel electrophoresis, then shifted onto PVDF membranes (Roche, Indianapolis, Indiana), after blocking of the membranes in 1 × 9 PBS and 0.1% Tween-20 containing 5% nonfat milk, then washed with PBS/Tween and membranes were incubated at 4°С for overnight with anti LYZL4 (1:1000, Bioss, Beijing, China), anti LYZL6 and anti PCNA antibody (1:1000, Aviva Systems Biology, San Diego, California) and anti β-actin (1:1000; Abcam, Cambridge, UK). The membranes were then incubated with a secondary antibody protein find goat anti rabbit IgG (H + L), HRP (Horseradish peroxidase) conjugate (1:5000; Trans Gen Biotech, Beijing, China) for 1 h, and Electrochemiluminescence (ECL) detection system (Pierce, Colorado) was used for visualization of the bands, the protein levels were measured by using densito metric analysis and finally the image was captured with a BA200Digital digital microscope (Motic China Group Ltd., Xiamen, China).

### Immunohistochemistry

Restore antigenicity of deparaffinized sections by autoclaving in a sodium citrate solution. Then, the slides were blocked into 5% serum in PBS at 25 °C for 1 h and incubated overnight at 4 °C with anti LYZL4 (1:500, Bioss, Beijing, China), anti LYZL6 and anti PCNA (1:500, Aviva Systems Biology, San Diego, California) antibodies, respectively. Testes sections of 2–3 um thickness were mounted on SuperFrost Plus microscope slides (Menzel-Gläser, Braunschweig, Germany), deparaffnized in xylol and rehydrated in a graded ethanol series. Antigen retrieval was induced by heat in 10 mM citrate buffer (pH 6.0) for 15 min at 100 °C. Thereafter, slides were placed in 0.3% hydrogen peroxide in methanol to quench endogenous peroxidases and blocked with 1.5% BSA and 10% horse serum. Incubation with the primary antibodies was carried out overnight at 4 °C. Signals were enhanced with the avidin/biotinylated peroxidase complex (Vectastain АBC Kit, Vector Laboratories, Burlingame, California), and color reactions were achieved by applying 3,3′-diaminobenzidine (DAB) as chromogen substrate (Liquid DAB + substrate Kit, Dako Schweiz AG, Baar, Switzerland). Finally, slides were counterstained with Mayer’s hematoxylin, dehydrated in a graded ethanol series and covered with coverslips. Isotype controls were carried out using pre-immune rabbit instead of the primary antibody. The integrated optical density of immunostaining for the LYZL4, LYZL6 and PCNA proteins was calculated by using Image-Pro Plus version 6.0 software (Media Cybernetics, Rockville, Maryland) to analyzed four random 400× microscope magnifications levels in sections.

### Statistical analysis

For gene expression levels, each experiment was repeated in at least three replicates, and the threshold cycle was calculated using the 2^−ΔΔC^*^t^* method.^22^ Data analysis was done by one-way ANOVA in SPSS version 21.0 software (SPSS, Chicago, IL). All data were expressed as ‘means ± SD’. A *p* value < 0.05 was established as a significant difference.

## Results

### The expression level of LYZL4, LYZL6 and PCNA mRNA by qRT-PCR

**Figure 1.**
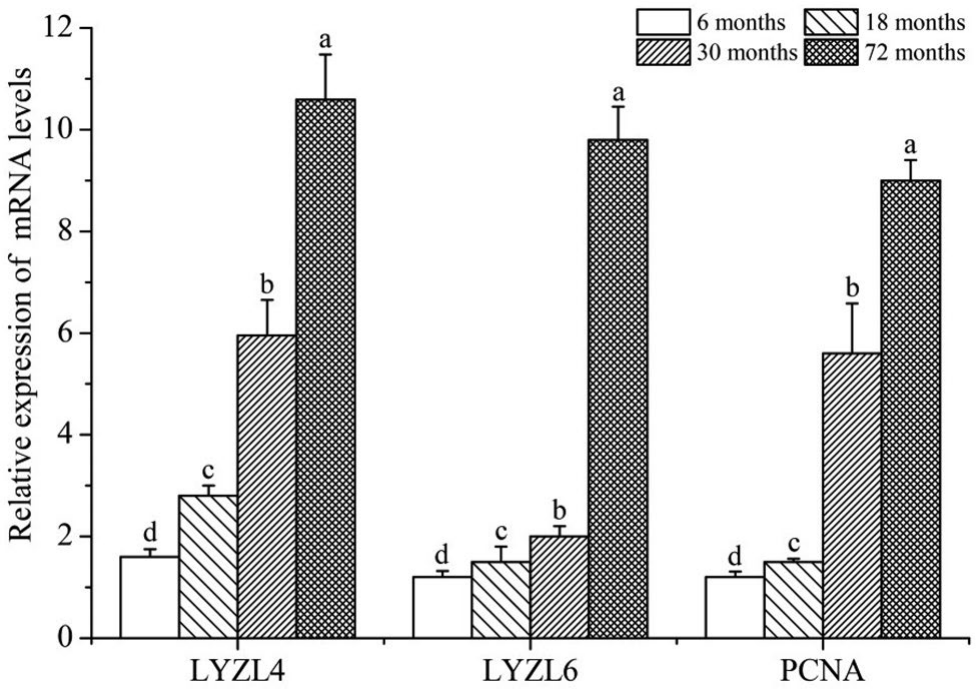
Relative expression of LYZL4, LYZL6 and PCNA mRNA in testis of the yak; different letters indicate significant difference (*p* < 0.01).

The expression of *LYZL4, LYZL6* and *PCNA* mRNA were evaluated by qRT-PCR at different stages of development in the yak. As shown in Figure 1, the expression of *LYZL4*, *LYZL6* and *PCNA* in the testis increased as yaks aged. *LYZL4*, *LYZL6* and *PCNA* were expressed in the yak testes at the four stages, with the highest level at 72 months (*p* < 0.01), followed by 30 months (*p* < 0.01), then 18 months (*p* < 0.01) and the lowest at 6 months (*p* < 0.01).

### Western blotting

**Figure 2.**
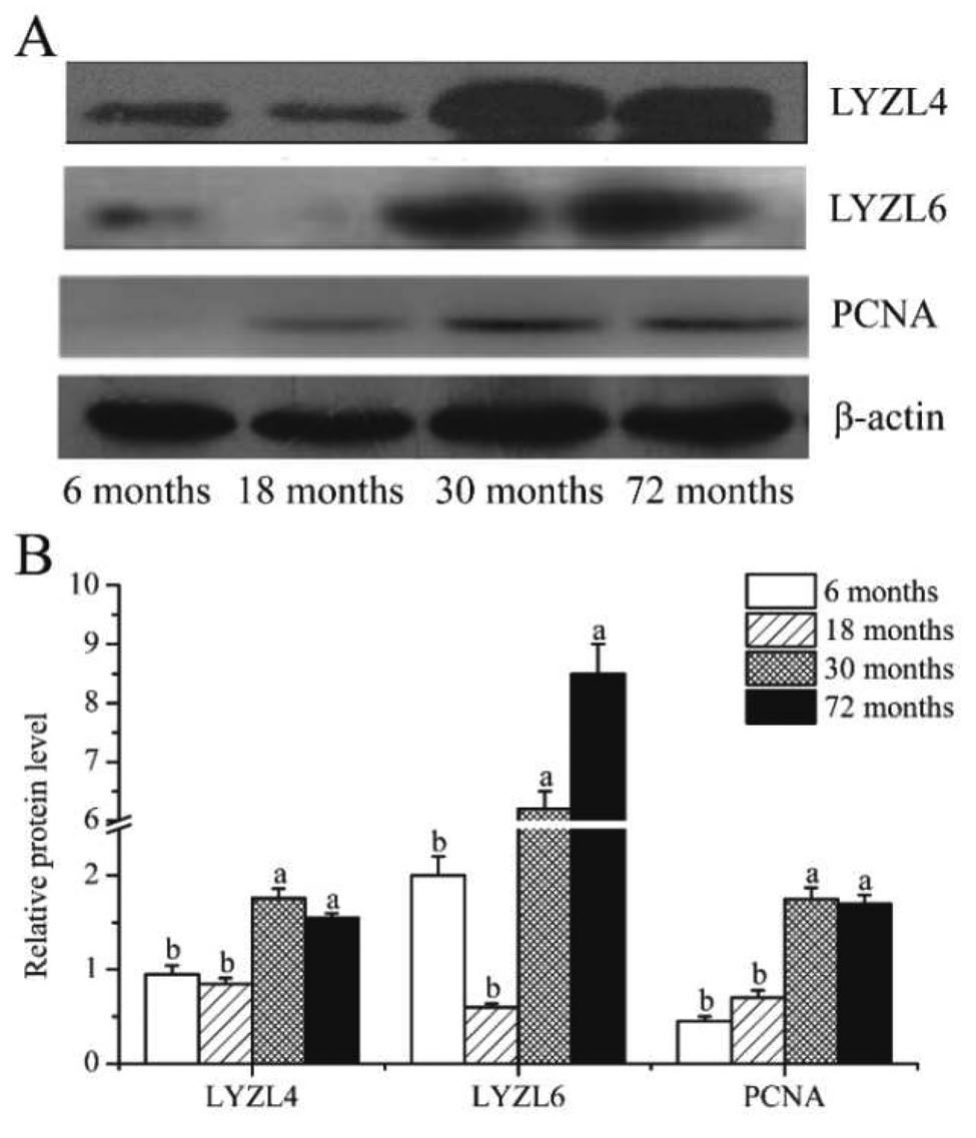
Expression of LYZL4, LYZL6 and PCNA protein in testis of the yak; (A) the LYZL4, LYZL6 and PCNA detected by Western blotting; (B) The protein expression of LYZL4, LYZL6 and PCNA; different letters indicate significant difference (*p* < 0.05).

We analyzed the protein expression features of LYZL4, LYZL6 and PCNA at different developmental stages of yak testes by Western blot. As shown in Figure 2, the LYZL4, LYZL6 and PCNA proteins were present in yak testes. The testes from the different ages showed no significant differences in the expression of LYZL4, LYZL6 and PCNA proteins between 30 and 72 months, but the expression levels in these two groups were significantly higher than at 6 or 18 months. Similarly, there was no significant difference in the expression of LYZL4, LYZL6 and PCNA proteins in the testes between 6 and 18 months.

### Immunohistochemistry for LYZL4, LYZL6 and PCNA

**Figure 3.**
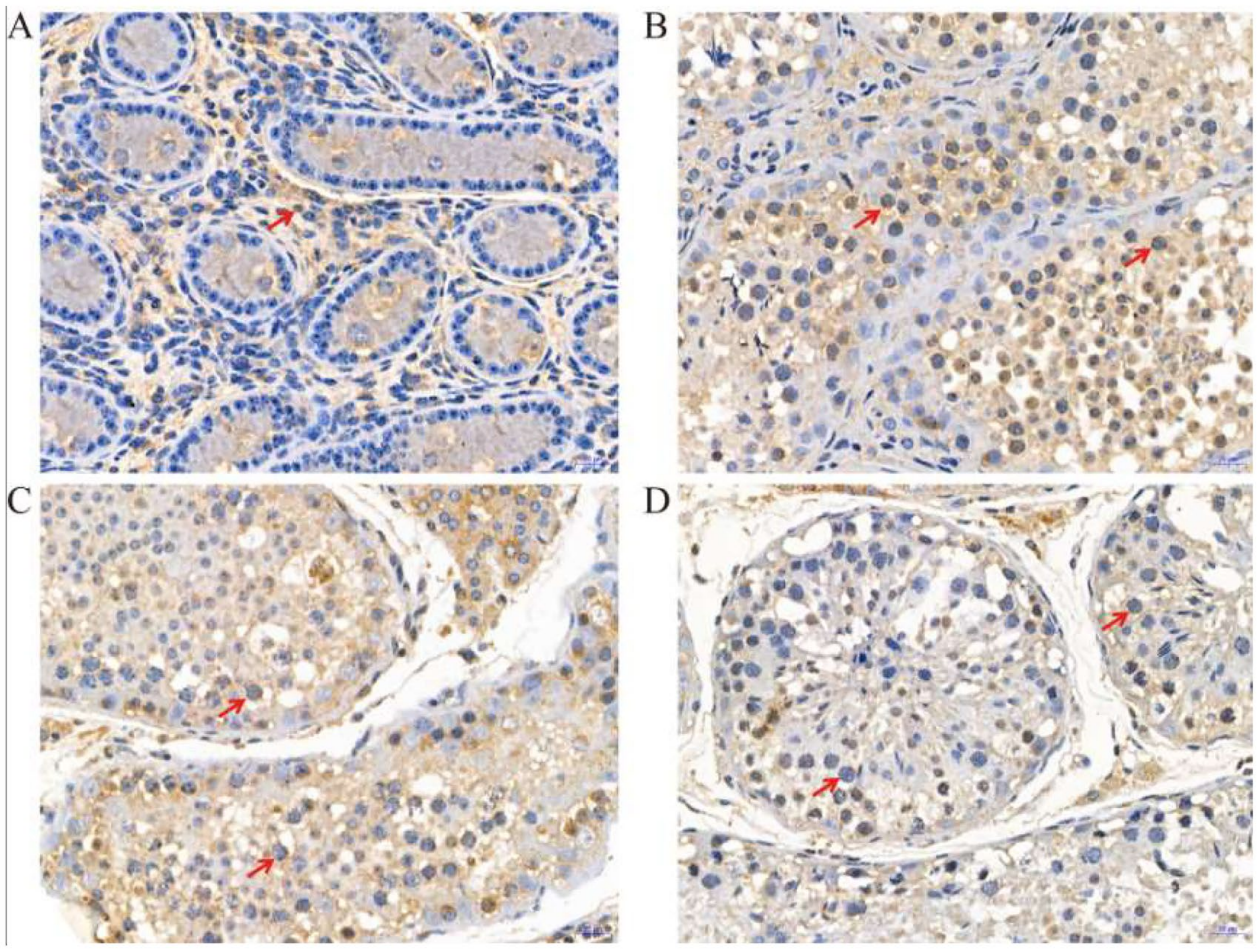
Immunohistochemical staining of LYZL4 protein at different developmental stages of yak testes (400×); (A) 6 months of Ashidan yak; (B) 18 months of Ashidan yak; (C) 30 months of Ashidan yak; (D) 72 months of Ashidan yak.

In addition, we used immunostaining analyses to study the LYZL4 LYZL6 and PCNA proteins at different developmental stages of yak testes. As shown in Figure 3, strong LYZL4 immunoreactivity was observed in Leydig cells at 6 months (Fig. 3A), intense positive reaction of LYZL4 was present in spermatogonia and Sertoli cells at 18 months (Fig. 3B) and intense positive reaction of LYZL4 was only present in round spermatids at 30 months (Fig. 3C) and 72 months (Fig. 3D).

**Figure 4.**
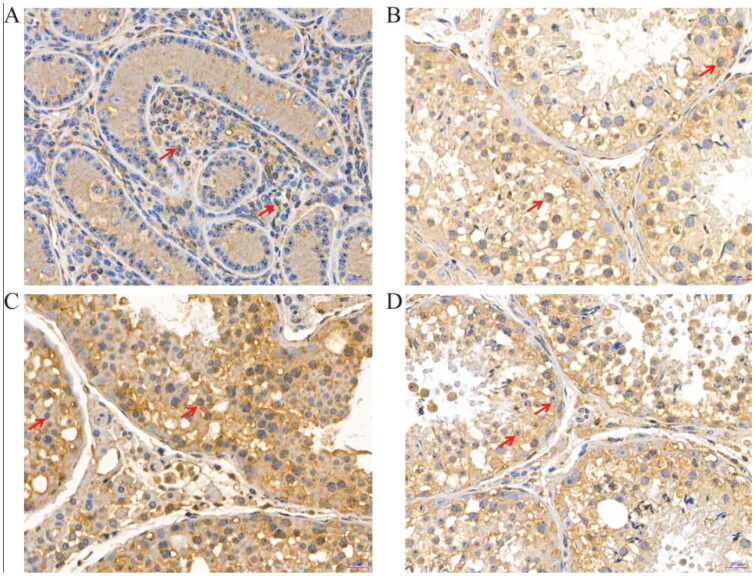
Immunohistochemical staining of LYZL6 protein at different developmental stages of yak testes (400×); (A) 6 months of Ashidan yak; (B) 18 months of Ashidan yak; (C) 30 months of Ashidan yak; (D) 72 months of Ashidan yak.

The results of immunohistochemical analysis of LYZL6 protein showed that strong LYZL6 immunoreactivity was observed in Leydig cells of yak testes at 6 months (Fig. 4A), and there was an intense positive reaction of LYZL6 in spermatogonia within the seminiferous tubules at 18 months (Fig. 4B). At the 30 (Fig. 4C) and 72 months (Fig. 4D), positive immunoreactions were observed in round spermatids and primary spermatocytes.

**Figure 5.**
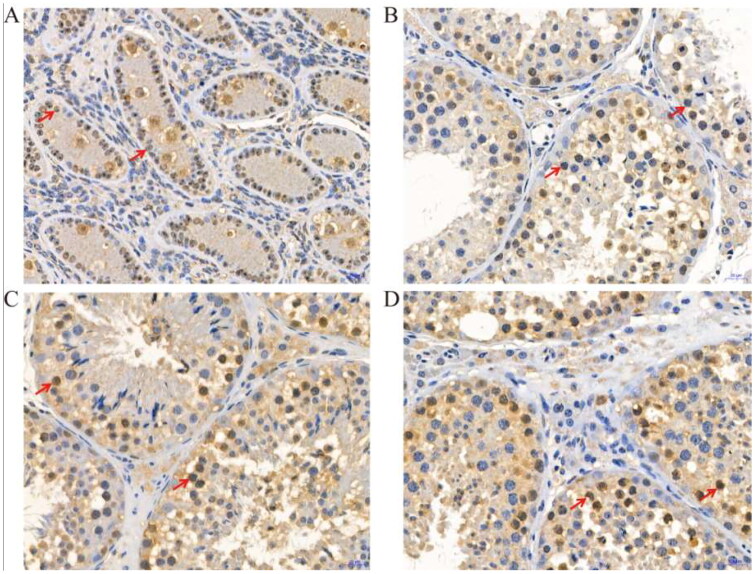
Immunohistochemical staining of PCNA protein at different developmental stages of yak testes (400×); (A) 6 months of Ashidan yak; (B) 18 months of Ashidan yak; (C) 30 months of Ashidan yak; (D) 72 months of Ashidan yak.

The results of immunostaining analyses of PCNA protein at different developmental stages of yak testes showed that at the 6 (Fig. 5A) and 18 months (Fig. 5B), epithelial cells and spermatogonia showed strong positive reactions. At the 30 (Fig. 5C) and 72 months (Fig. 5D), strong immunoreactivity was observed in round spermatids, primary spermatocytes and secondary spermatocytes.

## Discussion

Expression of proteins during the development of the testes is important to establish fertility in the adult animal. Studies have shown that many genes are involved in normal process of testicular development and spermatogenesis, but when these genes are mutated, they can cause spermatogenic disorder and infertility. The LYZL4 and LYZL6 are c-type lysozyme genes and they play crucial role in the normal development of testes and spermatogenesis in male animals.^23^ PCNA is a cell cycle regulatory protein marker, closely related to DNA synthesis, and involved in the proliferation and differentiation of spermatogonia in the testis.^24^ Although previous studies have shown that *LYZL4*, *LYZL6* and *PCNA* genes play crucial role in testicular development and spermatogenesis, but little is known about the role of *LYZL4*, *LYZL6* and *PCNA* genes in yak testes. Therefore, in this study, the *LYZL4*, *LYZL6* and *PCNA* genes expression level in different ages of yak testis was focused by testicular histology and morphology, as well as mRNA expression, protein abundance and immunohistochemical analysis of three candidate genes.

LYZL4 and LYZL6 are proteins with bacteriolytic activity and are widely expressed in various organ systems of many species, including fungi, plants, phages, birds, invertebrates and mammals.^25–27^ In humans, LYZL4 and LYZL6 are widely expressed in kidney, small intestine, lung, stomach, lymph node, lacrimal gland, spleen, parotid gland, placenta, sublingual gland and leukocytes, and the expression level in the testis are significantly higher than other organs.^8^^,^^28^^,^^29^ To further study the role of the LYZL family in reproduction, Wei et al. systematically studied the mRNA expression of LYZL family members in different murine tissues. Although LYZLs were expressed in other tissues, their expression in the testis and epididymis was significantly higher, which suggests that they have a potential role in the male reproductive tract.^12^ In this study, the results of the qPCR study showed that the mRNA expression levels of LYZL4 and LYZL6 in the yak testis increased significantly with the yak’s age. These results preliminarily confirmed that LYZL4 and LYZL6 may be involved in the development of yak testes. The expression of PCNA is closely related to cell division and proliferation, therefore, its expression changes are used as a marker for evaluating cell proliferation.^19^^,^^21^^,^^30^ In this study, through the qPCR study showed that the mRNA expression levels of PCNA in the yak testis increased significantly with the yak’s age. These results indicated that PCNA may be involved in the testicular development and spermatogenesis of yak.

Studies have shown that Western blot analysis holds several advantages over mRNA expression profiling with respect to identifying the function of genes.^31^ First, the presence of a specific mRNA in mammalian cells does not ensure that the protein is actually expressed.^32^ In addition, many proteins are activated or inactivated by post-translational modifications, which would not be detected by mRNA analysis.^33^ In order to better understand the functions of *LYZL4*, *LYZL6* and *PCNA* genes, we used Western blot analysis to detect the expression of LYZL4, LYZL6 and PCNA proteins at different developmental stages of yak testes. The results showed that at the age of 30 and 72 months, the expression levels of LYZL4, LYZL6 and PCNA proteins were significantly higher than those of 6 and 18 months. In this study, the protein and mRNA expression trends of LYZL4, LYZL6 and PCNA in yak testes of different ages were basically the same. These results further confirmed that LYZL4, LYZL6 and PCNA are involved in the development of yak testes. Furthermore, we used immunostaining analysis to study the morphological differences at different developmental stages of yak testes. LYZL4, LYZL6 and PCNA immunostaining were detected in yak testes at all development stages, mainly in the sperm cells. Huang et al. found that the immunoreactivity for LYZL4 in the acrosomal region of round and elongating spermatids of human.^34^ Sun et al. also found immunoreactivity for LYZL4 in the acrosomal region of round and elongating spermatids of mice.^23^ Narmadha et al. found that the rat LYZL4 and LYZL6 proteins are localized in the germinal epithelium and on the spermatozoa.^11^ Similarly, Wei et al. found that LYZL6 protein presence in primary spermatocytes and round spermatids of the testis and on the post-acrosomal area and midpiece of mature epididymal spermatozoa, suggesting that LYZL6 may contribute to the innate immunity of the male genital tract.^12^ Strong PCNA immunoreactivity has been observed in proliferating spermatogonia and spermatocytes in the testis of humans, cynomolgus monkeys, rhesus monkeys, and rats.^35–37^ Kang et al. detected strong PCNA immunopositive staining was located in the nuclei of spermatogonia in adult mice testis, while Verderame et al. detected strong PCNA immunopositive staining was found in rat testis only in spermatogonia.^38^^,^^39^ Similarly, in this study, immunostaining analysis found that LYZL4, LYZL6 and PCNA had strong immunoreactivity in Leydig cells and sperm cells, indicating that LYZL4, LYZL6 and PCNA are involved in the spermatogenesis and testicular development of yak, and suggesting that *LYZL4*, *LYZL6* and *PCNA* genes may affect yak reproduction by involving in spermatogenesis and testicular development. Our results of qPCR, western blotting and immunostaining analyses confirmed that the expression of the *LYZL4*, *LYZL6* and *PCNA* genes and their proteins increased with age in testes of yak and these proteins may be involved in the spermatogenesis and testicular development of yak.

## Conclusions

In this study, we found that the mRNA expression of *LYZL4*, *LYZL6* and *PCNA* genes in the testis increased with yak ages. In addition, western blotting was used to compare the content of LYZL4, LYZL6 and PCNA proteins at different developmental stages of yak testes. Consistent with our previous qPCR studies, amounts of LYZL4, LYZL6 and PCNA proteins in adult yak testes, detected by western blotting, were increased compared to those detected in prepubertal testes. Furthermore, the results of immunohistochemistry showed that the LYZL4, LYZL6 and PCNA proteins localization and the intensity of signals differed by age. Our results indicated that LYZL4, LYZL6 and PCNA may be involved in the development of Sertoli cells, Leydig cells and gonocytes in yak testes.
